# 

**Published:** 1920-07-10

**Authors:** 


### Forthcoming Events.

The State Children's Association, 53 Victoria Street, S.W. 1.—Annual meeting, Wednesday, July 14, at 3 o'clock, at 20 Portman Square (by the kind invitation of the Lord and Lady Islington), the Right Hon. the Earl of Lytton (Chairman of the Association) in the chair. Speakers: The Viscountess Astor, M.P., the Hon. Joan Poynder, the Right Hon. J. R. Clynes, M.P., and Mrs. S. A. Barnett, C.B.E. (Hon. Secretary).

The League of Mercy.—Her Royal Highness Princess Alice, Countess of Athlone, will receive the workers of the League of Mercy at a Garden Party at St. James's Palace on Friday, July 9.

Mr. Ernest E. Maddox, M.D., F.R.C.S.E., has resigned his appointment as ophthalmic surgeon to the Royal Victoria and West Hants Hospital, Bournemouth, on reaching the age limit. The warmest thanks of the Board were accorded him for his valued services during the past twenty-two years.

### Gifts and Bequests.

MR. SIMON FESTENSTEIN has left £20 to the Ear and Throat Hospital, Gray's Inn Road.

MRS. CHAVASSE, of Bromley, Kent, has bequeathed £20 to the Royal Hospital for Incurables.

MR. DAVID EVANS, of Swansea, has left £2,000 to the Swansea General and Eye Hospital for two "David Evans" beds.

MR. GEORGE L. MOORE, of Forest Hill, formerly in practice as a solicitor at Belfast, has intimated to the authorities of the Royal Victoria Hospital, Belfast, that it is his intention to give the institution £20,000 within two or three years. A scheme is being formulated for the establishment of a ward, to be called the "Moore Ward," in memory of a favourite sister of the donor.

AMONGST a number of bequests made by Mr. C. A. Heimann, of Sussex Gardens, W., are £2,000 to University College Hospital, to endow two beds in memory of his late wife; £1,000 each to the Hospital for Sick Children, Great Ormond Street, the Paddington Green Children's Hospital, and St. Mary's Hospital, to endow beds; and £1,000 each to Queen Charlotte's Hospital, Lord Mayor Treloar Cripples' Home; and £500 to the Italian Hospital for General Purposes.

